# Supplementary material for: The cross-sectional association between mean corpuscular volume level and cognitive function in Chinese over 45 years old: Evidence from the China Health and Retirement Longitudinal Study
Source: PLoS One. 2020 Dec 3;15(12):e0243227. doi: 10.1371/journal.pone.0243227 (PMC7714155; doi:10.1371/journal.pone.0243227)
Supplement: Attachment — Submitted filename: Response to Reviewers.docx [file pone.0243227.s002.docx]

Dear Dr. Suemoto:

In regards to our Manuscript PONE-D-20-25060R1 entitled **The cross-sectional association between mean corpuscular volume level and cognitive function in Chinese over 45 years old: evidence from the China Health and Retirement Longitudinal Study**, we would like to thank the reviewers and the Editors for their thorough, insightful and thoughtful review of our manuscript. Below, we have addressed each of the comments in a point-by-point manner. Changes to the text of the manuscript are highlighted in **BLUE** in the revised version of the manuscript.

Specially, both of the reviewers were concerned about table 5. We have used meta analysis to explore the potential heterogeneity (see below response to #2 reviewer in detail, our results suggested high heterogeneity existed for subjects with varied MCV level for both male and female subjects, this further supported that the association between MCV and cognitive function should be explored by sex. We think it might not be necessary to include this section of results in the final manuscript, and we have also removed table 5 per the first reviewer’s suggestion.

We feel the manuscript is much improved after revising based on the constructive comments from the reviewers and hope that you will find it acceptable for publication in Plos One.

Regards,

Zhongxiao Wan, PhD, Professor

Department of Nutrition and Food Hygiene

School of Public Health, Soochow University

Reviewers’ and editors’ comments are listed below, followed by our responses in bold-faced type.

Reviewer #1: In my opinion, the revision carried out by Wan and collaborators met the need for clarification, adjustment and improvement of methodology and analysis.
Stylistically, I personally prefer to just emphasize the central results and main tables of the study. Thus, optionally, all the section "Combined effect of MCV and sex on the prediction of cognitive performance" and table 5 can be eliminated. Leaving only described in the text this piece: “As a sensitivity analysis, we have utilized the multivariate logistic regression model to test the interaction term between sex and MCV. No combined effect was observed between MCV and sex. It is suggested that both sex and MCV affected cognitive function independently”.
My compliments to Wan and collaborators to considering our comments, doing a great revision work and an excellent final article.

**Response: Thank you very much! We have followed your suggestion, and removed table 5, with the results being described in the text. It should be mentioned that we have used meta analysis to explore the potential heterogeneity (see below response to #2 reviewer in detail), with heterogeneity were observed. This further confirmed that we should explore the association between MCV and cognitive function by sex.**

Reviewer #2: I'm fine with review which almost my comments.
The only final concern is how to reconcile the heterogeneity by sex in T4 with the evidence in T5 which suggest no heterogeneity, unless I missed something. In other words, the fully interacted model (T4) gives different results from the model with the sex interaction term (T5).

**Response: Thank you very much! We totally agree with your confusion, and we apologize for not solving this issue clearly in the first revision. In this revised manuscript, we have classified the subjects into 4 groups, i.e. female with 80≤MCV≤100 (low MCV), female with MCV>80 (high MCV), male with 80≤MCV≤100 (low MCV), and male with MCV>80 (high MCV). We have used meta analysis to explore the potential heterogeneity, I^2^<25% suggested little or no heterogeneity, 25–50% suggested moderate heterogeneity, and >50% was considered as high heterogeneity, respectively. As for Q statistic, p<0.1 indicated statistically signiﬁcant. As shown in below figure 1, our results suggested high heterogeneity existed for subjects with varied MCV level for both male and female subjects, this further suggested it is necessary to look at the association between MCV and cognitive function by sex. We have mentioned this part of results in the text and we have also removed table 5 per the first reviewer’s suggestion.**

Figure 1 Heterogeneity test via meta analysis.
